# Bridging Decades in Cutaneous Immunology—Past Lessons, Present Insights, Future Directions

**DOI:** 10.3390/biomedicines13112714

**Published:** 2025-11-05

**Authors:** Olga Simionescu

**Affiliations:** Department of Dermatology I, Colentina Clinical Hospital, Carol Davila University of Medicine, 020125 Bucharest, Romania; dana.simionescu@umfcd.ro

## 1. Introduction

The way in which our perception of the fundamental role played by the skin has changed is undoubtedly a hot topic. Initially viewed as a huge barrier due to its status as the largest organ in the human body (with a surface area of between 1.5 and 2 square metres), the skin is now recognised as an essential immune organ that orchestrates the interface between immunity and environmental factors through bidirectional host–environment interactions.

This change in perception is due to key molecular-level discoveries, which have enabled us to develop a more accurate understanding of the skin’s immune processes. This allows us to characterise skin disorders more accurately, including systemic autoimmune diseases that manifest in the skin.

## 2. Past Lessons: Early Immunodermatology (The Period Between 1960 and 1990)

The early study of immunology began against the backdrop of descriptive accounts of skin manifestations in lupus erythematosus. For example, Cazenave (1851) describes the skin lesions of lupus erythematosus, Kaposi presents “*erythema in vespertillo*” (1880), Hutchinson describes discoid lupus, and Sir William Osler contributes to our understanding of lupus erythematosus as a multisystemic disease. At that time, the numerous dermatological conditions were classified by Jean Louis Marc Alibert, a pioneer in the field who is known for his influential “arbre des dermatoses” (French for “tree of dermatoses”). And that is not all. He describes cutaneous T-cell lymphoma (mycosis fungoides), cells whose ascent from the dermis to the epidermis (“epidermotropism”) later becomes the key to histopathological diagnosis in routine HE staining.

Dermatology is primarily a morphological discipline because the skin can easily be accessed through biopsy. This period is characterised by the use of paraclinical methods for diagnostic confirmation, with histopathological examination being the dominant method (e.g., Gömöri silver impregnation is used to highlight the reticulin network in sarcoid versus Köster follicles in cutaneous tuberculosis).

Direct skin immunofluorescence (DIF), developed by Beutner and Jordan in 1963, became essential for characterising autoimmune bullous diseases [1] and photosensitivity dermatoses. Circulating anti-keratinocyte antibodies, pathogenic autoantibodies (1970), and new dermatoses (linear IgA disease, Chorzelski, 1978) are described. Genetic, non-immune, benign familial pemphigus, Hailey-Hailey (1939, Howard and Hugh Hailey), is differentiated from autoimmune pemphigus. DIF is negative in autosomal dominant forms and positive in autoimmune pemphigus, where acantholysis results from an immune response at different levels of the epidermis.

The “subepidermal” blister (actually, dermo-epidermal junction) defines bullous diseases produced without acantholysis through a distinct DIF profile. Almost 30 years after Walter Lever first described bullous pemphigoid, the condition was characterised by the immunoprecipitation of bullous pemphigoid antigens (BPAG2 and BPAG1).

Initial techniques in dermatological immunohistochemistry (IHC) can complement or replace DIF. Markers are used to differentiate between cutaneous lymphomas and pseudolymphomas, as well as between cutaneous T and B lymphocytes.

T cells soon became the focus of research, with significant findings regarding their activation in psoriasis and the subsequent loss of activation following phototherapy (PUVA) or cyclosporine treatment. These findings would later lead to the development of biological therapies. Biological therapies will later emerge from this seed.

The “brisk” and “non-brisk” cellular infiltrate are described in the histopathological profiling of cutaneous melanoma. The presence of this lymphocytic infiltrate is correlated with the host’s immune response to the tumour. From here, it is only a small step to redefining melanoma as an immunological disease by identifying the point at which it becomes an immunological tumour. Markers on lymphocytes (CD3, CD4) can be identified using monoclonal antibodies. The Th1 and Th2 lymphocyte subsets (described between 1980 and 1990) will be correlated with combined Gell–Coombs hypersensitivity mechanisms (types I and IV) in atopic dermatitis. Although B and T lymphocyte subpopulations had been described by 1960, the period from 1970 to 1980 is known as the “molecular revolution”.

Cytokines [2] are identified, and their role in human skin is elucidated: epidermal cells (keratinocytes) and dermal cells (lymphocytes) secrete proinflammatory cytokines while performing their own functions and interacting with each other. These simultaneous processes are highly accurate, with defence mechanisms being coordinated at the level of the skin in close connection with humoral and nervous mechanisms.

In 1973, Steinmann and Cohn, who both worked at Rockefeller University, identified a distinct population of cells in mouse lymphoid organs that they named *dendritic cells*. Consequently, Langerhans cells, which were first described by Paul Langerhans in 1868, are now considered immune cells rather than tissue macrophages. In the 1980s, skin dendritic cells were recognised as both *antigen-presenting cells* and triggers of the adaptive immune response through the activation of naïve T lymphocytes.

In inflammatory diseases, we noted the induction of new lesions upon trauma during that period (psoriasis vulgaris, lichen planus, vitiligo, and lupus erythematosus), which is clinically expressed by the Köbner phenomenon and is considered an immune response to trauma.

Atopic dermatitis is already considered an immunological disease mediated by immunoglobulin E IgE, the role of which in the development of the disease has been documented since 1966 (Kimishige & Teruko).

The introduction of “*Gell and Coombs*” hypersensitivity reactions in 1963 resulted in a redefinition of urticaria, as well as prompting new research into the roles of mast cells and the mechanisms of histamine release.

## 3. Present Insights (Last 30 Years)

Over the past 30 years, immunological research in dermatology has had a tremendous impact, leading to revolutionary treatments for inflammatory and autoimmune diseases, as well as skin cancer. Consequently, staging and treatment guidelines are being revised, as are the diagnostic criteria for conditions such as psoriasis, atopic dermatitis, melanoma, basal cell carcinoma, and pemphigus vulgaris.

New genetic discoveries and a better understanding of the molecular mechanisms of immunology in inflammatory skin diseases have redefined atopic dermatitis as “a genetic disease caused by filaggrin deficiency”, establishing a direct link between the epidermal barrier and inflammation.

Psoriasis is being reclassified from a simple genetic keratinisation disorder (genodermatoses) to a multifactorial polygenic disease. It is an autoimmune condition that occurs in the absence of autoantibodies and is proliferative without being tumourous in nature.

The classification of types I and II of psoriasis vulgaris is related to the presence of the class I HLA antigen HLA-C*06:02 (formerly HLA-Cw6), whose presence correlates with early onset, a family history of the condition, and an increased risk of arthritis. The HLA-C*06:02 genotype is also a predictive biomarker of the therapeutic response to psoriasis treatment [3].

TNF-α, IL-17A, and IL-23 have been characterised, and biological therapies targeting these have become routine for treating moderate to severe forms of psoriasis vulgaris and arthropathic psoriasis.

The role of cytotoxic T cells in alopecia areata is reinforced, as is the role of the following lymphocyte subtypes: Th1, Th2, Treg, Th9, and Th22. This improves our understanding of the mechanisms of inflammatory and autoimmune dermatoses.

Flow cytometry and scRNA-seq enable molecular analyses [4] that have an impact on modern advances in understanding innate and acquired immunity mechanisms.

Advances in dermatological genetics and epigenetics have led to the definition of cutaneous melanoma as a “malignant proliferation of melanocytes”, a genetic and immunological disease. Melanoma becomes an immunological tumour when micrometastases reach the “sentinel” lymph node. The presence of a primary malignant tumour in stage IV of the disease requires surgical excision, as it can secrete cytokines, growth factors, and exosomes that stimulate angiogenesis and create a “pre-metastatic niche” at a distant site.

The presence of advanced-stage melanoma can disrupt local and distant immune responses and generate severe local complications due to its role as a proliferative focus of active tumour cells.

Molecular diagnosis in melanoma specifies the types of mutations (BRAF V600, NRAS, c-Kit, NF1) and introduces liquid biopsy as a marker for detecting circulating tumour DNA (ctDNA), microRNA, or circulating tumour cells (CTDCs) [5].

Subsequently, the relationship between different types of melanoma and exposure to ultraviolet radiation is analysed, and two distinct categories of mutations in cutaneous melanoma are identified: genetic and acquired (spontaneous). Treatment guidelines for melanoma are updated according to the patient’s genetic profile for targeted therapy involving oral BRAF inhibitors (dabrafenib) or mitogen-activated protein kinase (MEK1 and MEK2) inhibitors (trametinib).

The soluble factors involved in innate skin immunity, as well as the drugs that target these factors, are described accurately [6].

*IHC* is advancing rapidly in the precise differentiation of dysplastic nevi with severe dysplasia from so-called Melanocytic Tumours of Uncertain Malignant Potential (MELTUMP) lesions or in situ melanoma. Tests that use monoclonal antibodies specific for melanoma (PRAME, SOX 10) are now common. These tests work with the classic analysis, which uses Ki-67, HMB45, and the S100 protein. The S100 protein is used as a “screening” tool to find melanocytes and metastases. However, it does not work as a prognostic or predictive marker.

### Biological and Targeted Therapies

In the early 2000s, general treatment with cyclosporine or methotrexate was replaced by the first biological dermatological therapies: TNF-alpha inhibitors, which are indicated for the treatment of moderate to severe arthropathic and vulgar psoriasis. Adverse reactions to these TNF-alpha inhibitors, such as tuberculosis, other infections, and malignancies, as well as in-depth research into immunological mechanisms, are paving the way for new biological therapies that target anti-CD11a, anti-IL12, 23, and 17. These therapies can achieve total clearance of psoriasis vulgaris, regardless of the trigger factor.

For chronic spontaneous urticaria, treatment protocols recommend omalizumab, an IgE inhibitor, to be prescribed after increasing doses of antihistamines or a combination of antihistamines and mast cell degranulation inhibitors have been exhausted.

Rituximab, a monoclonal antibody that expresses CD20 and targets B cells, is becoming the primary treatment for pemphigus vulgaris, a serious condition with a poor prognosis [7].

Significant progress is being made in the treatment of metastatic melanoma, with immunotherapy using checkpoint inhibitors having been shown to significantly prolong patients’ lives. One such inhibitor is nivolumab, a human monoclonal IgG4 antibody that blocks programmed death-1 (PD-1), a protein found on activated T lymphocytes, which promotes apoptosis and enhances the ability of T cells to recognise and destroy tumour cells.

Another IgG1 monoclonal antibody is ipilimumab, which targets cytotoxic T-lymphocyte antigen 4 (CTLA-4). This protein receptor is also expressed on T lymphocytes, thereby amplifying their activation and proliferation [8].

The neuro-immuno-cutaneous axis is becoming a hot topic in inflammatory diseases (atopic dermatitis, psoriasis, lichen planus), pruritic/psychodermatoses (excoriated acne, chronic pruritus [9], chronic spontaneous urticaria), vitiligo, and alopecia areata. Mast cells become activated and release inflammatory mediators. The function of keratinocytes and dendritic cells, as well as inflammation, is connected to the cutaneous nerve fibers via substance P or VIP (vasoactive intestinal peptide).

Apoptosis (programmed cell death) plays a role in lupus erythematosus, lichen planus and pemphigus vulgaris [10], carcinomas, and actinic pathology. Of the 12 types of cell death described in living organisms, it represents only one, alongside necroptosis (involved in psoriasis and atopic dermatitis), pyroptosis (in bacterial infections), and ferroptosis (in melanoma). In squamous cell carcinoma, levels of keratinocyte apoptosis increase in correlation with the p53 protein (“the guardian of the genome”).

Improving our understanding of acquired solar damage is fundamental to preventing skin cancer and premature ageing. Ultraviolet radiation (UVR) damages the DNA of keratinocytes, producing cyclobutane pyrimidine dimers, which are damage-associated molecular patterns (DAMPs). UVR also induces keratinocytes to release proinflammatory cytokines (IL-1, IL-6, and TNF-alpha) and activates Langerhans cells. In addition, the production of highly reactive oxygen species stimulates inflammatory pathways and destroys cellular DNA [11].

Another important area of research in skin immunology is the skin microbiome, which, in terms of diversity and abundance, ranks third after the gut and mucosal microbiomes. Since Nobel laureate Joshua Lederberg first described the microbiome in the 2000s, the skin has been seen not as a simple barrier, but as a nursery for microorganisms that play an immunological role.

The skin microbiome [12] educates the innate immune system via Toll-like receptors (TLRs), defensins and cathelicidins, and modulates acquired, adaptive immunity via Th1, Th2, Th17 and Treg lymphocytes. This helps to explain the need for skin hydration and the mechanisms of staphylococcal superinfection in patients with atopic dermatitis (AD).

The mechanisms of immune escape of *Treponema pallidum* (the causative agent of syphilis) [13] in *sexually transmitted diseases* (STDs) are precisely described, as are TROMPs, which serve as antigenic molecules capable of inducing immune responses via TLR-2. Gonococcal lipo-oligosaccharide (LOSs) stimulate innate immunity, resulting in the production of various cytokines (IL-6, IL-8, IL-1β, IL-17, and IFN-γ), which promote the chemotaxis and infiltration of polymorphonuclear leukocytes (PMNs) into the area of infection.

NAAT (nucleic acid amplification test) is becoming the primary method for diagnosing infections caused by *Chlamydia trachomatis* and *Neisseria gonorrhoeae*. It detects the genetic material (DNA or RNA) of the bacteria and is highly sensitive and specific.

Artificial intelligence is becoming an increasingly widely used tool in research and diagnostic methods, including IHC, digital imaging, proteomic analysis, skin microbiome analysis, and treatment prediction. It is fast and free from human error.

## 4. Future Directions—The Horizon +2025

**Figure 1:**
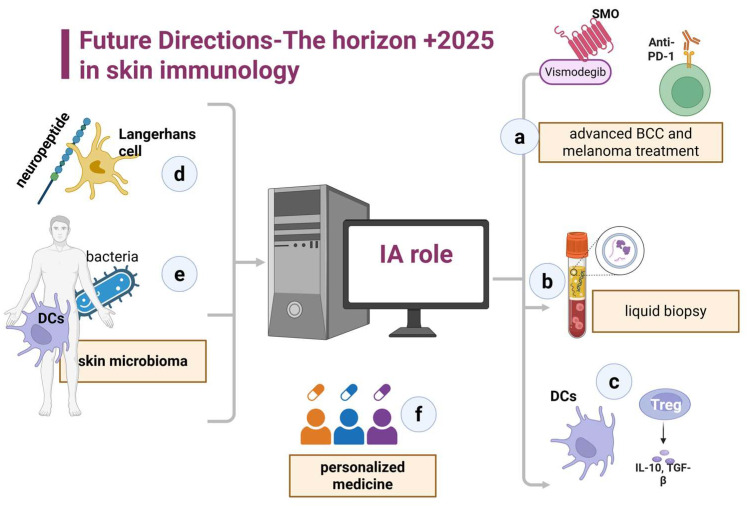
Future Directions—The horizon +2025 in skin immunology. (**a**) Differences in the action and response of hedgehog pathway inhibitors in carcinoma and PD1 inhibitors in melanoma. (**b**) A liquid biopsy is an example of a predictive marker for disease progression. (**c**) DCs and Tregs are possible candidates for future therapy. (**d**) The relationship between neuropeptides and skin immune cells. (**e**). Skin microbioma and dendritic cells. (**f**) A tailor-made immunological approach in dermatology is required.

In the coming years, skin immunology will advance rapidly within the field of translational medicine. Sophisticated screening and progressive diagnostic techniques will be used to address gaps in research and improve the understanding of dermatological conditions (Figure 1).

The response to therapy with Hedgehog pathway inhibitors in the treatment of advanced basal cell carcinoma (BCC) is weaker than the response to treatment with checkpoint inhibitors in melanoma. Further clarification is needed regarding secondary resistance (escape) after an initial positive response to treatment in severe cases of BCC. The mechanism of secondary mutations in neoplastic keratinocytes must be characterised. One possible explanation for the difference in response to treatment between carcinoma and melanoma is that checkpoint inhibitors target the acquired immune response.

Predictive markers of disease progression are needed in terms of their description, implementation, and accessibility. How quickly will liquid biopsies be incorporated into current practices and diagnostic and treatment protocols? In melanoma, liquid biopsies (blood-based biomarker assays) may be able to identify residual or recurrent disease before it can be seen on imaging studies. Together with circulating tumour cell (CTC) quantification from peripheral blood, it may provide superior information to lactate dehydrogenase (LDH), which is approved as a biomarker in advanced stages of melanoma but currently lacks very good sensitivity and specificity [14]. The BIOMAP consortium is analysing a series of biomarkers in patients with psoriasis that differ between the vulgar and arthropathic forms. These biomarkers include LCE3D, IL23R, IL23A, NFKBIL1, HLA-C*06:02, IL-17A, aHDL, GlycA, I-FABP, Kallikrein 8 and tyramine in the vulgar form, and HLA-C*06:02, HLA-B*27, HLA-B*38, HLA-B*08 and variation at the IL23R and IL13 loci, IL-17A, CXCL10, M2BP, integrin β5, matrix metalloproteinase-3, macrophage-colony stimulating factor, mucic acid and tyramine in the arthropathic form. These biomarkers require further validation [15].

A precise morpho-functional characterisation of immune population subtypes and an explanation for their lack of homogeneity are required. Progress has already been made in this field through the study of skin-associated lymphoid tissue (SALT) [16].

Will targeted therapies involving dendritic cells (DCs) and Tregs be available in the future? They show significant potential, with recent advances in genetic engineering, targeted antigens, and promising preclinical vaccines for antigen-specific Tregs and tailored DC vaccines. Treg therapies are focusing on genetic modification through next-generation genetic engineering.

Comorbid immune associations, such as Hashimoto’s autoimmune thyroiditis, alopecia areata, and psoriasis, are now explained by genetic factors, including variants of the IL-23 receptor gene. Common genetic susceptibility has also been reported in lichen planus and vitiligo, although different genetic loci may be involved. Damage to the immune system can cause a proinflammatory state, which facilitates the development of autoimmunity and cross-reactions of autoantibodies. In the future, it is likely that biomarkers will be used for screening and predicting associations [17].

What is the precise nature of the relationship between neuropeptides and skin immune cells? It has now been demonstrated that neuropeptides influence the production, activation, migration, and release of immune cells, which has practical applications in wound healing.

What is the relationship between the skin microbiome and dendritic cells and lymphocytes? How can we manipulate the microbiome? In chronic dermatoses involving microbiome imbalances (skin/intestinal), there is potential for remodelling of DC subpopulations, hence the enthusiasm for microbiome-modulating therapies such as microbiota transplantation and probiotics.

A superior understanding of the mechanisms governing the duration of biological therapy with a given product is required when treating biologically naive patients or switching between biological therapies. Different access depending on geography and different ways of reimbursing costs through healthcare systems could be a limitation.

The treatment of chronic immune-mediated inflammatory dermatoses could be improved if we were able to establish the role of adaptive immunity in disease relapse and the capacity of the innate immune system for memory of individual cells. This memory is mediated by epigenetic mechanisms [18].

What is the connection between localised scleroderma (morphea, LoS) and systemic scleroderma (SS)? The word “mysterious” that currently defines this relationship differentiates LoS and SSc as two distinct clinical entities that may coexist [19], but with the potential to transition from the cutaneous form to the systemic form. The reason why morphea and other autoimmune skin diseases (e.g., chronic lupus erythematosus, psoriasis and atopic dermatitis) do not have circulating autoantibodies remains to be clarified. Is it simply an expression of T-cell-mediated autoimmunity with locally generated autoantibodies? What causes the lack of penetration into the systemic circulation, especially since soluble immunoglobulins are involved? What role do genetic, environmental, or inferred autoimmune processes play?

Can we expect to benefit from personalised medicine for skin immunity in the near future? How accessible will individual genetic and cytokine profiles be for identifying the most appropriate treatment? Chimeric antigen receptor (CAR) T cells are genetically engineered T lymphocytes that improve overall survival for patients with large B-cell lymphoma and progression-free survival for patients with multiple myeloma. Could CAR-T cell therapy [20].

Finally, what will the real impact of AI be on diagnosis and treatment? Will AI continue to support medical staff, or will it replace them, given the incredible advances in laboratory and clinical diagnostics, imaging, and research tools? Who will be responsible for the medical aspects, given that AI requires careful validation, ethical consideration of data privacy, and human oversight?

## 5. Discussion

A superior understanding of skin immunology has led to significant progress in treating autoimmune diseases affecting the skin, as well as skin cancer. We would never have understood the multisystemic nature of lupus erythematosus if Kaposi had not first described erythema *in vespertillo*.

Progress has been made since the first steps in classifying skin lesions and introducing the first immune diagnostic methods in understanding the role of T lymphocytes, their subsets, and how proinflammatory cytokines are released by T lymphocytes and keratinocytes. Following the early period of immunodermatology, exceptional advances have been made in this medical specialty over the last 30 years. Furthermore, new genetic discoveries are changing our understanding of atopic dermatitis, psoriasis, and skin melanoma.

Previous techniques are being modernised, and new methods of paraclinical investigation are emerging. Biological and targeted therapies are becoming standard in care protocols developed worldwide.

The concept of the skin neuroimmune axis and the skin microbiome is clarified, as is the role of apoptosis in carcinogenesis mechanisms. The mode of action of bacteria in sexually transmitted infections (STIs) is better understood in this significant public health issue.

In the coming years, both clinical dermatologists and researchers will need to address the real challenges of the present. These include providing a more accurate description of the predictive markers of progression and the different responses to immune therapy in carcinoma versus melanoma. Other challenges include exploring the potential use of dendritic cells in targeted therapies of the future and investigating the relationship between neuropeptides and skin immune cells.

There is also the implementation of personalised medicine to consider, which is tailored to the individual patient, not to mention the role of artificial intelligence in diagnosis and treatment.

## 6. Conclusions

Skin immunology is a sophisticated field of research that has transformed our view of the human skin, shifting the focus from a simple barrier to an orchestrator of the interface between immunity and environmental factors through the lens of bidirectional host-environmental factor interaction.

This editorial aims to connect past lessons (early immunodermatology) with present insights (of the last 30 years), demonstrating developments in the field and highlighting knowledge gaps and future directions (up to the year 2025). This bridge across time shows us that progress in the diagnosis, staging, and treatment of personalised medicine is supported by the immunology of the largest organ in the human body: the skin.

## Abbreviations

The following abbreviations are used in this manuscript:

DIF	Direct Skin Immunofluorescence
BPAG1	Bullous Pemphigoid Antigen 1
BPAG2	Bullous Pemphigoid Antigen 2
IHC	Immunohistochemistry
PUVA	Psoralen Ultraviolet A
CD	Cluster of Differentiating Antigens
Ig	Immunoglobulin
HLA-C*06:02	Human Leukocyte Antigen *06:02
HLA-Cw6	Human Leukocyte Antigen Cw6
TNF-α, IL	cytokines
Th	T helper cell
T reg	Regulatory T cell
scRNA-seq	Single-Cell RNA Sequencing
BRAF	B-Raf proto-oncogene, serine/threonine kinase
NRAS	Neuroblastoma RAS viral oncogene homolog
c-Kit	CD117
ctDNA	NF1 circulating tumour DNA
CTDCs	circulating tumour cells.
MEK	Mitogen-Activated Protein Kinase
MELTUMP	MELanocytic Tumour of Uncertain Malignant Potential.
PRAME	PReferentially expresses Antigen in MElanoma
SOX10	SRY-related HMG-box protein 10
Ki-67	protein involved in cell division and proliferation
HMB45	Human Melanoma Black 45
PD-1	Programmed death-1 protein
CTLA-4	Cytotoxic T-lymphocyte antigen 4
VIP	Vasoactive intestinal peptide
UVR	Ultraviolet ray
DAMPS	Damage/Danger-Associated Molecular Patterns
TLRs	Toll-like Receptors
STD	Sexually transmitted diseases
TROMPs	Treponema Outer Membrane Proteins
LOSs	Gonococcal lipo-oligosaccharide
IFN-γ	Interferon γ
PMNs	Neutrophils
NAAT	Nucleic acid amplification test
BCC	Basal cell carcinoma
CTC	Circulating tumour cell
LDH	Lactate dehydrogenase
BIOMAP consortium	Biopharmaceutical Manufacturing Preparedness Consortium
LCE3D	Late Cornified Envelope Family in Differentiating Epithelia
NFKBIL1	NF-kappa-B inhibitor-like protein 1
HDL	High-Density-Lipoprotein
GlycA	Glycosylated Acute-Phase Proteins
I-FABP	Intestinal Fatty Acid-Binding Protein
CXCL10	C-X-C motif chemokine 10
SALT	Skin-associated lymphoid tissue
DCs	Dendritic cells
LoS	Localised scleroderma
SS	Systemic scleroderma
(CAR) T cells	Chimeric Antigen Receptor for T cells
